# Resolvin D1 attenuates CCl4 Induced Liver Fibrosis by Inhibiting Autophagy-Mediated HSC activation *via* AKT/mTOR Pathway

**DOI:** 10.3389/fphar.2021.792414

**Published:** 2021-12-20

**Authors:** Jiahuan Li, Xiaoling Deng, Shuhan Wang, Qianqian Jiang, Keshu Xu

**Affiliations:** ^1^ Department of Infectious Diseases, Union Hospital, Tongji Medical College, Huazhong University of Science and Technology, Wuhan, China; ^2^ Division of Gastroenterology, Union Hospital, Tongji Medical College, Huazhong University of Science and Technology, Wuhan, China

**Keywords:** resolvin D1, liver fibrosis, autophagy, HSC activation, Akt/mTOR pathway

## Abstract

Resolvin D1 (RvD1) was previously reported to relieve inflammation and liver damage in several liver diseases, but its potential role in liver fibrosis remains elusive. The aim of our study was to investigate the effects and underlying mechanisms of RvD1 in hepatic autophagy in liver fibrosis. *In vivo*, male C57BL/6 mice were intraperitoneally injected with 20% carbon tetrachloride (CCl4, 5 ml/kg) twice weekly for 6 weeks to establish liver fibrosis model. RvD1 (100 ng or 300 ng/mouse) was added daily in the last 2 weeks of the modeling period. *In vitro*, lipopolysaccharide (LPS)-activated LX-2 cells were co-treated with increasing concentrations (2.5–10 nM) of RvD1. The degree of liver injury was measured by detecting serum AST and ALT contents and H&E staining. Hepatic fibrosis was assessed by masson's trichrome staining and metavir scoring. The qRT-PCR, western blot, immunohistochemistry, and immunofluorescence were applied to liver tissues or LPS-activated LX-2 cells to explore the protective effects of RvD1 in liver fibrosis. Our findings reported that RvD1 significantly attenuated CCl4 induced liver injury and fibrosis by decreasing plasma AST and ALT levels, reducing collagen I and α-SMA accumulation and other pro-fibrotic genes (CTGF, TIMP-1 and Vimentin) expressions in mouse liver, restoring damaged histological architecture and improving hepatic fibrosis scores. *In vitro*, RvD1 also repressed the LPS induced LX-2 cells activation and proliferation. These significant improvements mainly attributed to the inhibiting effect of RvD1 on autophagy in the process of hepatic stellate cell (HSC) activation, as demonstrated by decreased ratio of LC3-II/I and elevated p62 after RvD1 treatment. In addition, using AZD5363 (an AKT inhibitor that activates autophagy) and AZD8055 (an mTOR inhibitor, another autophagy activator), we further verified that RvD1 suppressed autophagy-mediated HSC activation and alleviated CCl4 induced liver fibrosis partly through AKT/mTOR pathway. Overall, these results demonstrate that RvD1 treatment is expected to become a novel therapeutic strategy against liver fibrosis.

## Introduction

Liver fibrosis is the final common pathological condition of chronic liver injury of various etiologies and closely associated with cirrhosis, liver failure, and hepatocellular carcinoma (Parola and Pinzani, 2019). Acute and self-limiting fibrosis is generally regarded as a reversible, protective response to liver tissue injury. However, the persistent injurious stimuli in the liver gives rise to the accumulation of extracellular matrix (ECM) components, which largely disrupt the normal architecture and functioning of the liver, thus liver fibrosis irreversibly deteriorates to advanced cirrhosis with life-threatening consequences (Kisseleva and Brenner, 2021). Hepatic stellate cells (HSCs) are dominant contributors to fibrogenesis in the injured liver. Previous studies have shown that activated HSCs could transdifferentiate into myofibroblasts, which further produce amounts of main components of ECM, including collagen type I and α-smooth muscle actin (α-SMA) (Roehlen et al., 2020). In addition, activated HSCs secrete transforming growth factor-β1 (TGF-β1) to induce more quiescent HSCs activation and promote ECM deposition, initiating a positive feedback loop to accelerate the progress of liver fibrosis (Dewidar et al., 2019). Although it has been clear that the inhibition of HSCs activation and ECM deposition is crucial for blocking hepatic fibrogenesis, there is still limited efficacious antifibrotic pharmacologic treatments available. Hence, identifying and delivering effective therapies to counteract liver fibrogenic progression are urgently needed.

Autophagy is an essential and highly conserved intracellular compounds degradation pathway mediated by lysosomal machinery and plays an important role in the evolution of eukaryotes (Yu et al., 2018). In the liver, apart from regulating normal metabolic functions such as glycogen decomposition, glycogen generation and β -oxidation, autophagy is also involved in liver diseases caused by various factors (Weiskirchen and Tacke, 2019; Ueno and Komatsu, 2017). Recent studies have claimed that autophagy may act as a critical profibrogenic mechanism involved in liver fibrosis (Hernández-Gea et al., 2012; Li et al., 2018). Mediated through degrading intracellular lipids and retinol into free fatty acid, autophagy provides energy that allows quiescent HSCs to transform into a myofibroblast-like phenotype (Hernández-Gea and Friedman, 2012; Park et al., 2018). In addition, 3-methyladenine (3-MA), an autophagy inhibitor, has been proven efficient in inhibiting the proliferation and activation of HSCs, as well as attenuating liver fibrosis (Wang et al., 2017). Moreover, lipopolysaccharide (LPS), an activator from intestinal flora, was demonstrated to promote autophagosome formation and increase autophagic flux in LX-2 cells and HSCs through the AKT-MTOR and AMPK-ULK1 pathway (Chen et al., 2017). Thus, the activation of autophagy is considered to be necessary for HSCs action and blocking autophagy in HSCs might be a promising therapeutic strategy against liver fibrosis.

**FIGURE 1:**
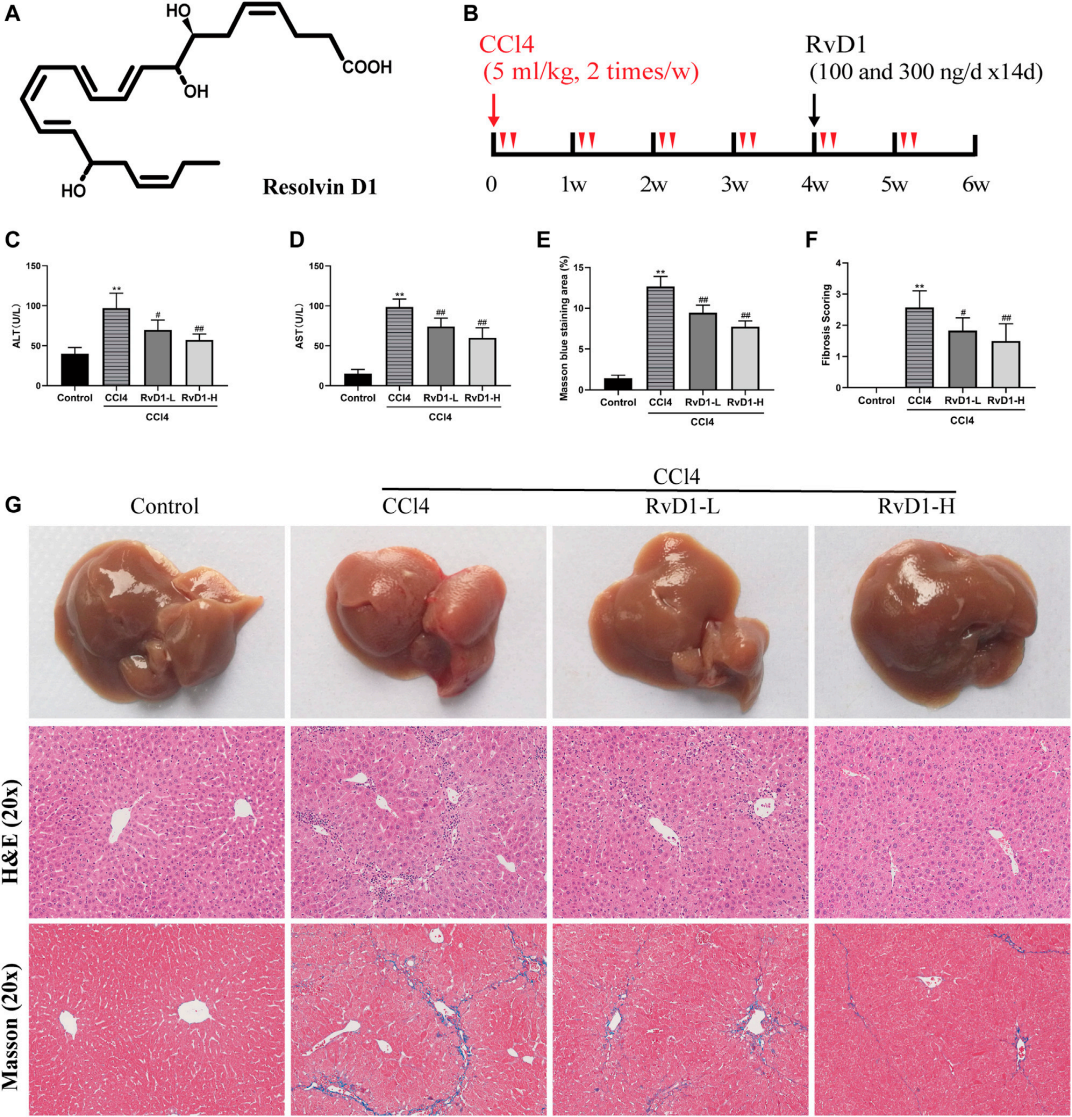
RvD1 attenuated liver injury and fibrosis in CCl4-treated mice. The mice were divided into four groups (Control, CCl4, RvD1-L, RvD1-H) and treated as described in the “Materials and methods” section. **(A)** Chemical structure of RvD1. **(B)** Animals experimental design. **(C and D)** Serum levels of ALT and AST in each group. **(E)** Metavir score of liver samples. **(F)** Semi-quantitative analysis of the collagen fibre staining (blue staining areas) is shown. **(G)** Representative images of gross morphology, H&E and Masson staining in liver samples (magnification: 20×, scale bar: 200 μm). Data are presented as the mean ± SD, n = 5–6 mice/group. *p < 0.05, **p < 0.01 vs. the Control group; ^#^ *p* < 0.05, ^##^ *p* < 0.01 vs. the CCl4 group.

Resolvin D1 (RvD1, 7S,8R,17S-trihydroxy-4Z,9E,11E,13Z,15E, 19Z-docosahexaenoic acid (DHA), molecular formula, C22H32O5, Figure 1A) is the main bioactive metabolite converted from the omega-3 polyunsaturated fatty acid DHA (Buckley et al., 2014; Chiang and Serhan, 2020). DHA possesses a wide range of biological functions, including anti-inflammatory, antioxidant, anti-tumor activity, hepatoprotection, renoprotection, and neuroprotection (Cardoso et al., 2016; Yang et al., 2019). As DHA-derived mediator, RvD1 exerts strong antioxidant and anti-inflammation properties and has been shown safe and protective in liver diseases through various pathways (Jung et al., 2014; Zhang et al., 2015; Li et al., 2020). It is demonstrated that RvD1 potently relieves carbon tetrachloride (CCl4)-induced hepatotoxicity in mice by inhibition of oxidative stress and inflammation (Chen et al., 2016). In addition, RvD1 has been found to protect the liver from ischemia/reperfusion injury by enhancing M2 macrophage polarization and efferocytosis (Kang and Lee, 2016). Our previous studies have also suggested that RvD1 improves hepatic dysfunction in methionine-choline deficient (MCD)-diet induced steatohepatitis (Li et al., 2020). However, whether RvD1 has beneficial effects in liver fibrosis remains unclear, as well as the underlying mechanisms. Recent studies showed that RvD1 exerted anti-fibrotic activity in lung and kidney interstitial tissue by inhibiting abilities of proliferation and collagen synthesis in fibroblast (Qu et al., 2012; Zheng et al., 2018). Since myofibroblasts transdifferentiate from activated HSCs in the liver, there is a possibility that RvD1 may reverse liver fibrosis by acting on HSCs.

It was reported that RvD1 relieved autophagy damage and restored autophagy flux by reducing the production of autophagy related markers and the formation of autophagy vacuoles, thus alleviating cerulein-induced acute pancreatitis (Wang et al., 2018). While, Patricia Prieto et al. found that RvD1 induces the formation of MAP1LC3(+) autophagosomes in macrophages and promotes the fusion of autophagosome and lysosome (Prieto et al., 2015). Moreover, RvD1 enhances phagocytic activity of macrophages through the regulation of nuclear factor, erythroid 2 like 2 (NFE2L2), and improves the survival ability and function of macrophages (Prieto et al., 2015). The above evidences suggest that RvD1 has different regulatory effects on autophagy in different disease models and cells. However, whether RvD1 can regulate autophagy in HSC cells remains unknown.

Here, to explore the effect and potential mechanisms of RvD1 in hepatic fibrosis, both a CCl4-induced hepatic fibrosis murine model and human LX-2 cells were treated with RvD1. We found that RvD1 improved liver damage and fibrosis partly by inhibiting autophagy in HSCs. Our current study suggests that RvD1 may be a potential therapeutic option for ameliorating hepatic fibrosis or other chronic fibrotic disease associated with dysregulated autophagy.

## Materials and Methods

### Reagents and Antibodies

RvD1 (HPLC ≥ 95%, BR, FW: 376.5, CAS Registry No.: 872993-05-0) was supplied by Cayman Chemical Company (Ann Arbor, United States). CCl4 (C8029) and olive oil (O8111) were obtained from Wuhan Promoter Biological CO., LTD. (Wuhan, China). LPS (L2630) and Cell Counting Kit 8 (CCK-8; CK04) were from Sigma-Aldrich (Merck KGaA, Darmstadt, Germany) and Dojindo Molecular Technologies (Shanghai, China), respectively. AZD5363 (AKT inhibitor, SF2820) and AZD8055 (mTOR inhibitor, SC0042) were provided by Beyotime Biotechnology Co., LTD. (Shanghai, China). The specific primary antibody information for western blot is shown in Supplementary Table S1. Secondary anti-mouse/rabbit antibodies conjugated with horseradish peroxidase (HRP, western blot) were provided by Proteintech Group, Inc (Rosemont, IL, United States). Green fluorescence-labeled antibodies (Alexa Fluor 488) and red fluorescence-labeled antibodies (Alexa Fluor 594) were products of Jackson Immuno Research, Inc (Baltimore, PA, United States). Fetal bovine serum (FBS), Lipofectamine™ 3000 (L3000015) and Opti-MEM (A4124801) were purchased from Gibco (Waltham, MA, United States).

### Cell Culture and Treatment

LX-2 cells (human hepatic stellate cell line) were purchased from Procell Life Science&Technology Co., Ltd. (Wuhan, China) and used to validate the vivo data. LX-2 cells were seeded in Dulbecco's modified Eagle's medium (DMEM) (containing 10% FBS) and grown under standard cell culture conditions (5% CO_2_, 37°C). RvD1 (100 nM) was dissolved in ethanol and stored at −80°C. When the cell grows to the logarithmic stage, they were planted into 6-well plate or 12-well plate at 1 × 10^5^/well or 1 × 10^6^/well for PCR or western blot detection. Different concentrations of LPS (1, 10, 100 ng/ml) were used to determine the optimal concentration to induce LX-2 cells activation. And a series of increasing concentrations of RvD1 (2.5, 5, 10 nM) were used to treat LX-2 cells. The dosage of RvD1 used *in vitro* was based on previous studies with minor modifications (Jung et al., 2014; Isopi et al., 2020). Each treatment was measured in triplicate.

### Animals and Treatments

Male 8 week-old C57BL/6 J mice weighing 21–25 g were provided by Vital River Laboratory Animal Technology Co., Ltd (Beijing, China). After a week of adaptation, mice were treated with vehicle or CCl4 for 6 weeks and co-treated with RvD1 at the last 2 week of the experiment. CCl4 was dissolved in olive oil at a ratio of 1:4 (v/v) and RvD1 (100 ng or 300 ng) was dissolved in 0.2 ml phosphate buffer saline (PBS) immediately before use. The experimental design was outlined in Figure 1B. The animals were divided into four groups (6 mice per group) as follows: (I), control group: mice received olive oil by intraperitoneal injection twice weekly for 6 weeks (Control); (II), model group: mice received 5 μl per gram body weight (B.W.) of a CCl4/olive oil mixture by intraperitoneal injection twice weekly for 6 weeks to induce liver fibrosis (CCl4); (III), low-dose RvD1 intervention group: as model group but mice received 100 ng RvD1 daily via intraperitoneal injection for 2 weeks, starting after 4-week treatment of CCl4 (RvD1-L); (IV), high-dose RvD1 intervention group: as model group but mice received 300 ng RvD1 daily via intraperitoneal injection for 2 weeks, starting after 4-week treatment of CCl4 (RvD1-H). The dosage design of RvD1 was based on our previous study, which is considered as a therapeutic dose in mice without obvious toxicity (Li et al., 2020). We also did preliminary experiment and confirmed that RvD1 treatment alone did not change normal pathological morphology of liver (Supplementary Figure S1). During the injection period, animals were kept in a specific pathogen-free (SPF) environment well ventilated with sufficient food and water, in 12 h day/night cycle. Ethical approval was obtained for the experiment from the Institutional Animal Care and Use Committee of Tongji Medical College, Huazhong University of Science and Technology.

At the end of 6 weeks, mice were sacrificed with pentobarbital sodium and the blood samples were collected and centrifuged (3,000 rpm, 15 min) to obtain serum for analyzing the effect of RvD1 on biochemical parameters. Livers of the mice were collected and weighed. Some specimens were used immediately or frozen at −80°C for the detection of molecular biological changes while other parts were immersed in 4% paraformaldehyde for histopathological evaluation.

### Serum Biochemistry

Biochemical assays (C010-2-1 and C009-2-1, Nanjing Jiancheng Bioengineering Institute) were used to measure serum alanine aminotransferase (ALT) and aspartate aminotransferase (AST) activity, respectively. The absorbance of each blood sample was read at 510 nm wavelength and raw optical density (OD) were converted into liver enzyme activity according to the instructions.

### H&E and Masson’s Trichrome Staining

Fresh liver tissues approximately 0.5 cm^2^ in size were fixed with 4% paraformaldehyde, embedded in paraffin, and then cut into 4 μm-thick sections. Hematoxylin and eosin (H&E) and masson’s trichrome staining were performed in the thin sections as described previously to evaluate liver injury and the changes of liver fibrosis (Feng et al., 2018). The tissues were imaged by Olympus BX-51 microscope (Tokyo, Japan) at 20× magnification. The degree of liver fibrosis was graded based on the metavir score, which semi-quantitatively classify each case from 0 to 4: a score of 0 expressed no fibrosis, four expressed cirrhosis, and 1-3 expressed enlarged portal tract with varying degrees of septa (Rousselet et al., 2005; Raizner et al., 2017). The degree of liver fibrosis was quantified and analysed with ImageJ V1.8.0 software (National Institutes of Health).

### Immunohistochemistry Analysis

For IHC analysis, the paraffin-embedded liver sections (4 μm) were pretreated with dewaxed, rehydrated and heat-induced antigen retrieval. Then, the sections were immersed in hydrogen peroxide (3%) for 10 min to neutralize endogenous peroxidase activities and sequentially incubated with goat serum (5%) for 30 min to block non-specific binding sites of the tissues. Next, after immunostaining overnight at 4°C with antibodies against collagen I (1:500; Cat. no. GB11022-1), α-SMA (1:500; Cat. no. GB13044), the slides were immunostained with a biotinylated secondary antibody (1:100; Cat. no. G1216) for 1 h at room temperature. The primary antibodies and biotinylated secondary antibody used in IHC staining were purchased from Wuhan Servicebio Technology Co., Ltd (Wuhan, China). DAB chromogenic reagent (AR1022; Boster; Wuhan, China) was added to visualize positive staining and hematoxylin (AR0005; Boster; Wuhan, China) was used to mark the nucleus. For semi-quantitative analysis of positive cells, images (20× magnification) were acquired by microscopy and analysed using ImageJ V1.8.0 software (National Institutes of Health).

### Quantitative Real-Time Polymerase Chain Reaction

Total RNA was extracted from LX-2 cells and liver tissues using the RNAiso Plus reagent (Cat. no. 9109; Takara; Beijing, China) and reverse transcribed to complimentary DNA (cDNA) with Prime Script RT reagent kit (RR036A; Takara; Beijing, China) using 1 μg of purified total RNA as a template. TB Green fluorescent quantitative PCR kit (RR420A; Takara; Beijing, China) was used for the quantification of the mRNA expression of Connective tissue growth factor (CTGF), tissue inhibitor of metalloproteinase (TIMP)-1, Vimentin, α-SMA, Collagen I, ULK1, ATG5, Beclin1, ATG7, ATG9A and GAPDH using the Light Cycler 480 software (Roche Diagnostics GmbH, Mannheim, Germany). The reaction procedure was as follows: 1) denaturation stage: 95°C, 15s; 2) annealing stage: 60°C, 30 s; 45 cycles; 3) extension stage: 95°C, 5s. The primers used in qRT-PCR assay were synthesized by Tsingke Biological Technology (Beijing, China), which sequences are summarized in Supplementary Table S2. The target gene levels were measured based on their cycle threshold (Ct) values and analysed by the 2^−ΔΔCt^ method. GAPDH was used as reference genes.

### Protein Extraction and Western Blot Assay

Total protein lysates were obtained from LX-2 cells and frozen mouse liver tissues by incubation on ice with RIPA lysis buffer containing 1% phenylmethanesulfonyl fluoride (PMSF) and cocktail inhibitor. After centrifugation at 12,500 rpm for 15min at 4°C, the supernatant was collected, followed by quantification of the protein amount with a bicinchoninic acid (BCA) kit (P1511-2; Applygen; Beijing, China). Then the protein samples mixed with 5x sodium dodecyl sulfate (SDS) protein loading buffer were denatured at 100°C for 10 min. Equivalent amounts of denatured proteins were separated on 10% SDS-polyacrylamide gels (P0012A; Beyotime; Shanghai, China) by western blot and immediately transferred to polyvinylidene difluoride (PVDF) membranes (HVLP02500; Millipore; Billerica, MA, United States). Next, the blots were incubated with skimmed milk powder (5%) for 1 h, followed by specific primary antibodies listed in Supplementary Table S1 at 4°C overnight, and the respective secondary antibodies (1: 2,000 dilution) at room temperature for at least 1 h. Finally, immunoreactive strips were revealed with an enhanced chemiluminescence (ECL) kit (ANT044; Antgene; Wuhan, China).

### Cell Viability Assay

For measuring cell proliferation, a proportion of LX-2 cells (1 × 10^4^ cells/ml) were plated into 96-well plates. After 24 h of culture, the various concentrations of RvD1 (1.25, 2.5, 5, 10 nM) and 100 ng/ml LPS were added to the culture medium for another 24 h. Cells in control group were treated with an equal volume of PBS. After incubation of 10 μl CCK-8 reagent for 2 h, the absorbance at 450 nm of each well was measured by a microplate reader to assess the effect of RvD1 on cell growth.

### Immunofluorescence

To monitor autophagic activity, immunofluorescence examinations were carried out to detect the expression of microtubule-associated protein 1A/1B-light chain 3B (LC3B) and SQSTM1/p62. Briefly, 4 μm paraffin-embedded liver sections were soaked in dimethylbenzene and graded ethanol for dewaxing and then were put into heated citrate buffer (pH 6.0) for antigen retrieval. Next, the slices were treated with 0.3% Triton X-100 and blocked with 1% donkey serum, followed by incubation with a mixture of anti-LC3B (Sigma, 1:100) and anti-p62 (Proteintech, 1:200) primary antibodies at 4°C overnight. On the following day, the liver sections were incubated with appropriate Alexa Fluor 488 and 594-conjugated secondary antibodies (1:500) for 1 h at room temperature and stained with 1 μg/ml DAPI (C1002; Beyotime; Shanghai, China) for 5 min. After exhaustive washing in PBS, the sections were eventually visualized with a laser confocal scanning microscope (Olympus, FV-500, Tokyo, Japan). For the cell experiment, LX-2 cells were seeded into 24-well plates and then transfected with GFP-mRFP-LC3 reporter plasmids (Yingrun Biotechnologies Inc., China) using Lipofectamine™ 3000 and Opti-MEM for 24 h before treatment with RvD1 and LPS. Then, after fixation with 4% paraformaldehyde, the cells were photographed using the Olympus FV-500 confocal microscopy (Tokyo, Japan). Fluorescent spots in at least 10 cells of each group were counted and all sets of cell experiment were repeated at least three times.

### Statistical Analysis

Data were expressed as the mean ± standard deviation (SD). Differences between groups were determined by one-way analysis of variance (ANOVA) in GraphPad Prism 8.0.1 (GraphPad Software Inc., La Jolla, CA, United States). *p* < 0.05 was considered significant.

## Results

### RvD1 Attenuated Liver Injury and Fibrosis in CCl4-Treated Mice

To examine the protective effect of RvD1 on liver injury and fibrosis, C57BL/6 mice were injected intraperitoneally with either olive oil or CCl4 for 6 weeks. Liver sections from the CCl4 group displayed widespread proliferated fibrous tissue in the portal triads and massive hepatocellular edema/necrosis around central veins, with hepatic cord disappearance and pseudolobules formation, as evidenced by H&E and masson staining. Herein, our study established a successful liver fibrosis animal model. Surprisingly, RvD1 treatment effectively repaired the destruction of liver tissue structure and reduced collagen fiber hyperplasia (Figures 1E,G), thus the fibrosis scoring was correspondingly decreased according to pathophysiological evaluations (Figure 1F). Consistent with the histological findings, ALT and AST, two biochemical diagnostic markers of hepatocellular damage, were also significantly decreased with the RvD1 dose in CCl4-induced mice (Figures 1C,D). In addition, although there was no significant difference observed in mice liver weight, the mice body weight returned concomitant with decreased liver index after RvD1 intervention (Table 1). These results suggested that RvD1 exerted a significant relieving effect on liver injury.

**TABLE 1:** Body weight, liver weight and liver index of mice in each group.

Index	Control	CCl4	RvD1-L	RvD1-H
body weight (g)	25.40 ± 1.192	20.73 ± 2.171**	23.04 ± 1.946^#^	24.47 ± 1.056^##^
liver weight (g)	1.119 ± 0.097	1.078 ± 0.108	1.122 ± 0.152	1.109 ± 0.102
liver index	0.044 ± 0.003	0.052 ± 0.003**	0.049 ± 0.003	0.045 ± 0.004^##^

Control mice received olive oil by intraperitoneal injection, CCl4 mice received intraperitoneal injections of CCl4/olive oil mixture (5 ml/kg, 2 times/w) for 6 weeks, and RvD1 mice were subjected to CCl4 intervention and received RvD1 administration (RvD1-L: 100 ng, RvD1-H: 300 ng) by intraperitoneal injection. Liver index = liver weight/body weight; Data are expressed as the mean ± SD, n = 5–6 mice/group.*p < 0.05, **p < 0.01 vs. the Control group; #p < 0.05, ##p < 0.01 vs. the CCl4 group, n = 5–6 mice/group

### RvD1 Inhibited HSC Activation and ECM Formation in CCl4-Treated Mice

**FIGURE 2:**
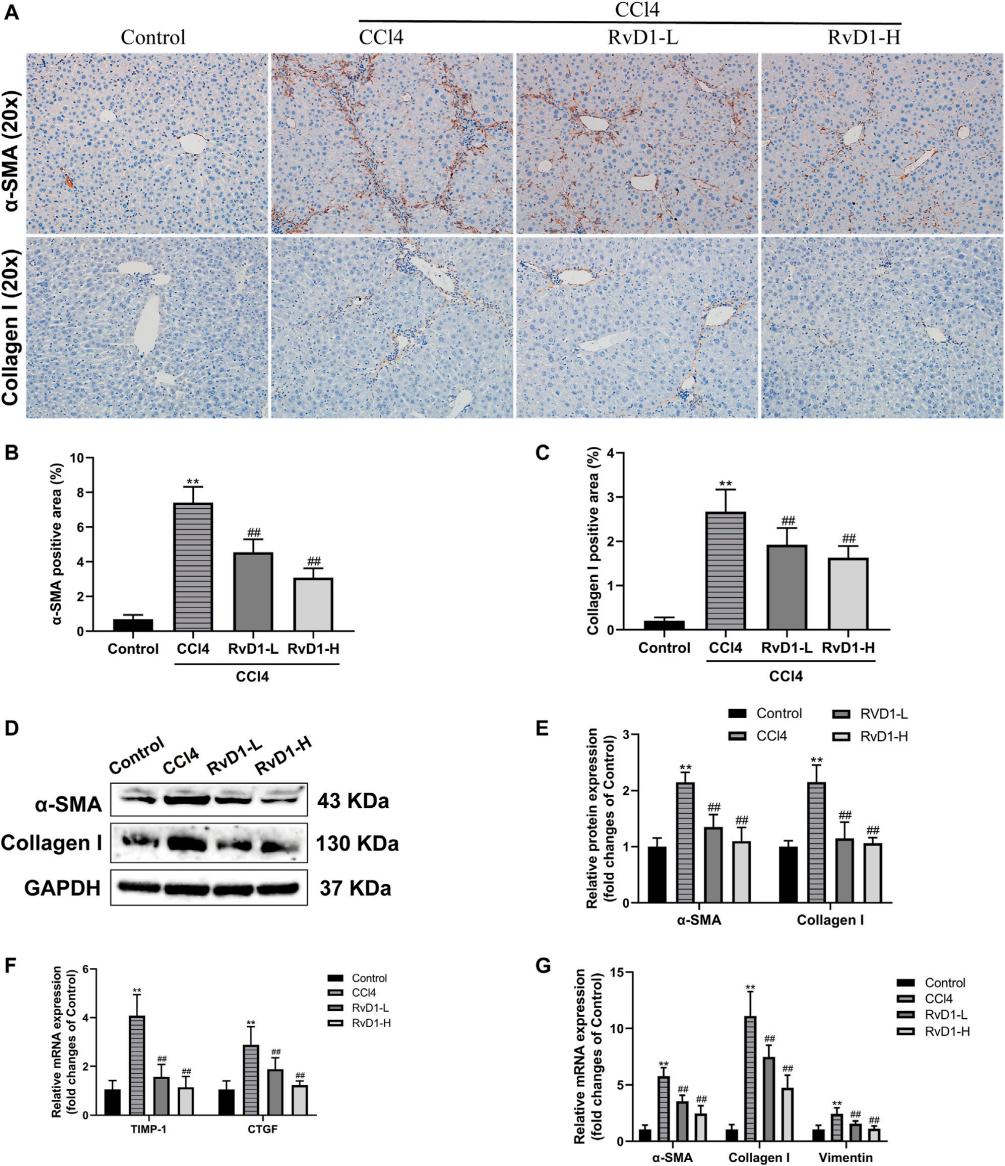
RvD1 inhibited HSC activation and ECM formation in CCl4-treated mice. **(A)** Representative images of α-SMA and collagen I immunohistochemical (IHC) staining in liver samples. **(B and C)** Semi-quantitative analysis of α-SMA and Collagen I IHC-positive staining areas is shown. **(D and E)** The protein levels of α-SMA and Collagen I were evaluated by western blot in mouse liver tissues from the indicated groups. Representative bands are shown. **(F and G)** The qRT-PCR analysis of CTGF, TIMP-1, Vimentin, α-SMA and Collagen I mRNA levels in mouse liver samples. Data are presented as the mean ± SD, n = 5–6 mice/group. *p < 0.05, **p < 0.01 vs. the Control group; ^#^ *p* < 0.05, ^##^ *p* < 0.01 vs. the CCl4 group.

Next, we investigated the effects of RvD1 on hepatic fibrosis and HSC activation. α-SMA is a biomarker of activated HSC, while Collagen I is as the main component of ECM (Tsuchida and Friedman, 2017). As shown in Figure 2, results from western blot and IHC detection showed that compared with the control group, α-SMA and Collagen I in the liver tissues of mice were significantly increased after CCl4 injection, while these two proteins were significantly reduced in a dose dependent manner following the administration of RvD1 (Figures 2A–E), indicating that RvD1 could significantly inhibit HSC activation and ECM formation. Apart from α-SMA and Collagen I, we also detected the expression of various fibrotic factors. CTGF, as a common downstream cause of fibrotic stimulation, is significantly increased when the liver is damaged, and plays an important role in mediating liver fibrosis (Makino et al., 2018). The main role of TIMP-1 is to inhibit metalloproteinases to reduce the degradation of ECM and promote collagen deposition (Wang et al., 2013). Vimentin is expressed in almost of all mesenchymal cells and its main function is to maintain the integrity of cytoskeleton. Therefore, the expression of Vimentin is commonly significantly increased after HSC converted into myofibroblasts (Wang et al., 2019). Compared with the Control group, obvious increases in mRNA levels of α-SMA, Collagen I, CTGF, TIMP-1 and Vimentin in mouse liver tissues in CCl4 model group was observed. However, these changes were partially reversed in a dose dependent manner after RvD1 intervention (Figures 2F,G).

### LPS Induced the Activation and Proliferation of LX-2 Cells

**FIGURE 3:**
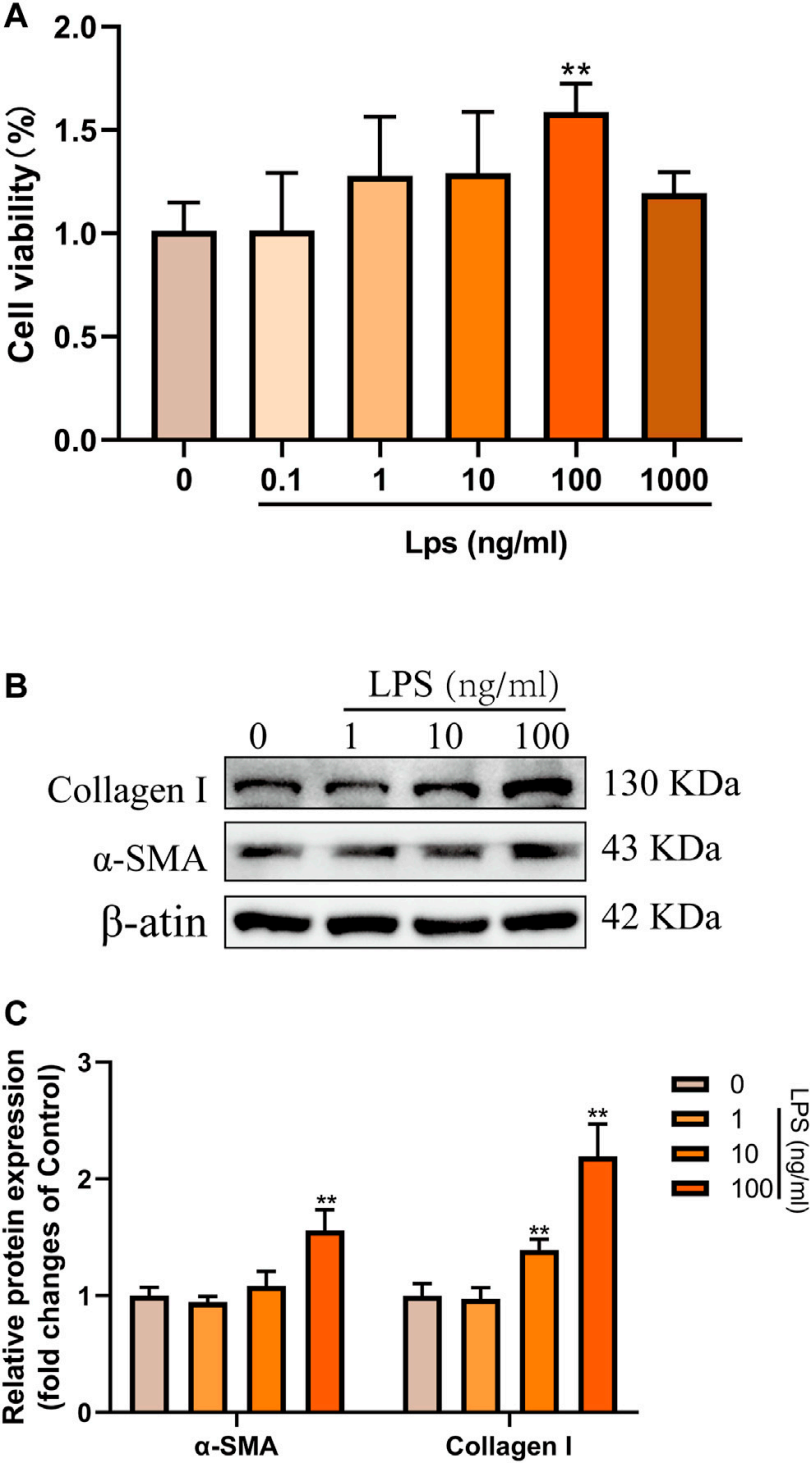
LPS induced the activation and proliferation of LX-2 cells. **(A)** LX-2 cells were treated with the increased concentrations (0.1–1,000 ng/ml) of LPS or an equal volume of PBS for 24 h. CCK8 assays showed proliferation change of cells (n = 6 samples/group). **(B and C)** Western blot analysis of Collagen I and α-SMA protein levels in LX-2 cells activated by different concentrations (0–100 ng/ml) of LPS. At least three independent experiments were carried out. Data are presented as the mean ± SD, n = 4 samples/group. *p < 0.05, **p < 0.01 vs. the Control group.

Next, LPS (0.1–1,000 ng/ml) was used to induce the activation of LX-2 cells to determine the optimal concentration for simulating the process of liver fibrosis *in vitro*. The effects of LPS on the proliferation of LX-2 cells were detected by CCK-8 kit. The results showed that the proliferation of LX-2 cells was significantly increased at 100 ng/ml LPS, compared with the blank control group (*p* < 0.01) (Figure 3A). However, when the concentration of LPS was 1,000 ng/ml, the viability of LX-2 cells decreased (Figure 3A), which may be because prolonged high-dose LPS stimulation results in excessive cytotoxic effects in turn promotes more cells apoptosis (Yang et al., 2013). Accordingly, as demonstrated by western blot, we found that the protein expressions of α-SMA and Collagen I were significantly increased in LX-2 cells treated with LPS at the concentration of 100 ng/ml (Figures 3B,C). Therefore, 100 ng/ml LPS was used for subsequent experiments.

### RvD1 inhibited LX-2 Cell Proliferation, Activation and ECM Production

**FIGURE 4:**
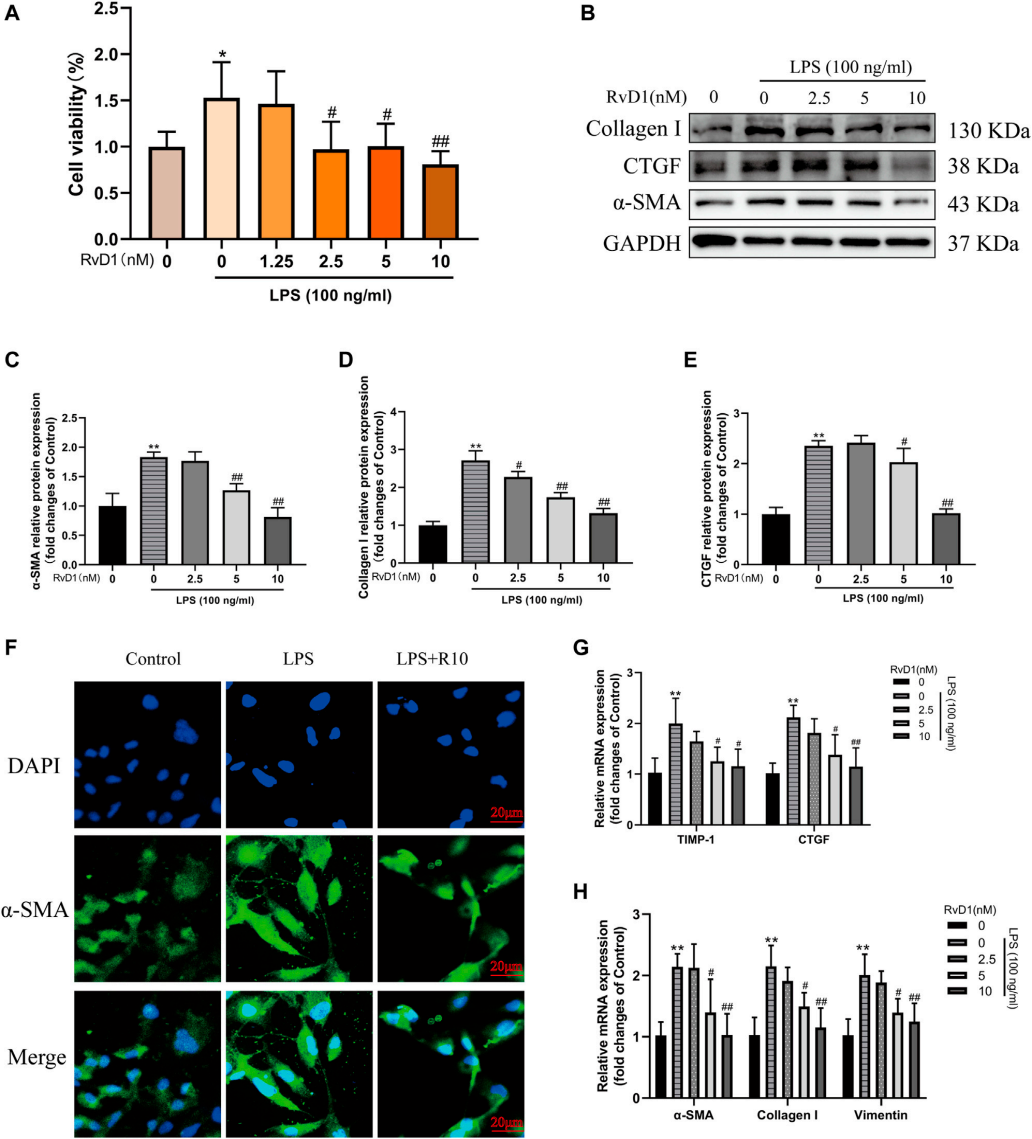
RvD1 inhibited LX-2 cell proliferation, activation and ECM production. **(A)** LX-2 cells were co-treated with the indicated concentrations (1.25–10 nM) of RvD1 and LPS (100 ng/ml) for 24 h. Cells in control group received PBS treatment. CCK8 assays showed proliferation change of cells. **(B–E)** Western blot analysis of Collagen I and α-SMA protein levels in LX-2 cells treated with indicated concentrations (1.25–10 nM) of RvD1 and LPS (100 ng/ml) for 24 h. **(F)** Representative images of immunofluorescent staining against α-SMA (green color) in LX-2 cells, scale bar = 20 μm. Cells in LPS + R10 group received 10 nM RvD1 and 100 ng/ml LPS treatments for 24 h **(G and H)** The qRT-PCR analysis of CTGF, TIMP-1, Vimentin, α-SMA and Collagen I mRNA levels in LX-2 cells. At least three independent experiments were carried out. Data are presented as the mean ± SD, n = 4 samples/group. *p < 0.05, **p < 0.01 vs. the Control group; ^#^ *p* < 0.05, ^##^ *p* < 0.01 vs. the LPS group.

Subsequently, LX-2 cells were treated with both 100 ng/ml LPS and RvD1 at different concentrations (1.25–10 nM) to investigate the effect of RvD1 on the proliferation of HSCs. It was found that with the increase of RvD1 concentration, the viability of LX-2 cells decreased in a dose dependent manner (Figure 4A). Similarly, consistent with the *in vivo* results, the protein and mRNA levels of activated HSC marker α-SMA were also significantly decreased in a dose dependent manner with the increase of RvD1 concentration (Figures 4B,C,F,G), as well as Collagen I and CTGF (Figures 4B–H). The decreased mRNA expression levels of pro-fibrotic related genes TIMP-1 and Vimentin in LX-2 cells following RvD1 treatment further supported the anti-fibrosis activity of RvD1 *in vitro* (Figures 4G,H). The above results verified that RvD1 had an inhibitory effect on LX-2 cell proliferation, activation and ECM production.

### RvD1 Alleviated CCl4-Induced Liver Fibrosis in Mice by Regulating Autophagy

**FIGURE 5:**
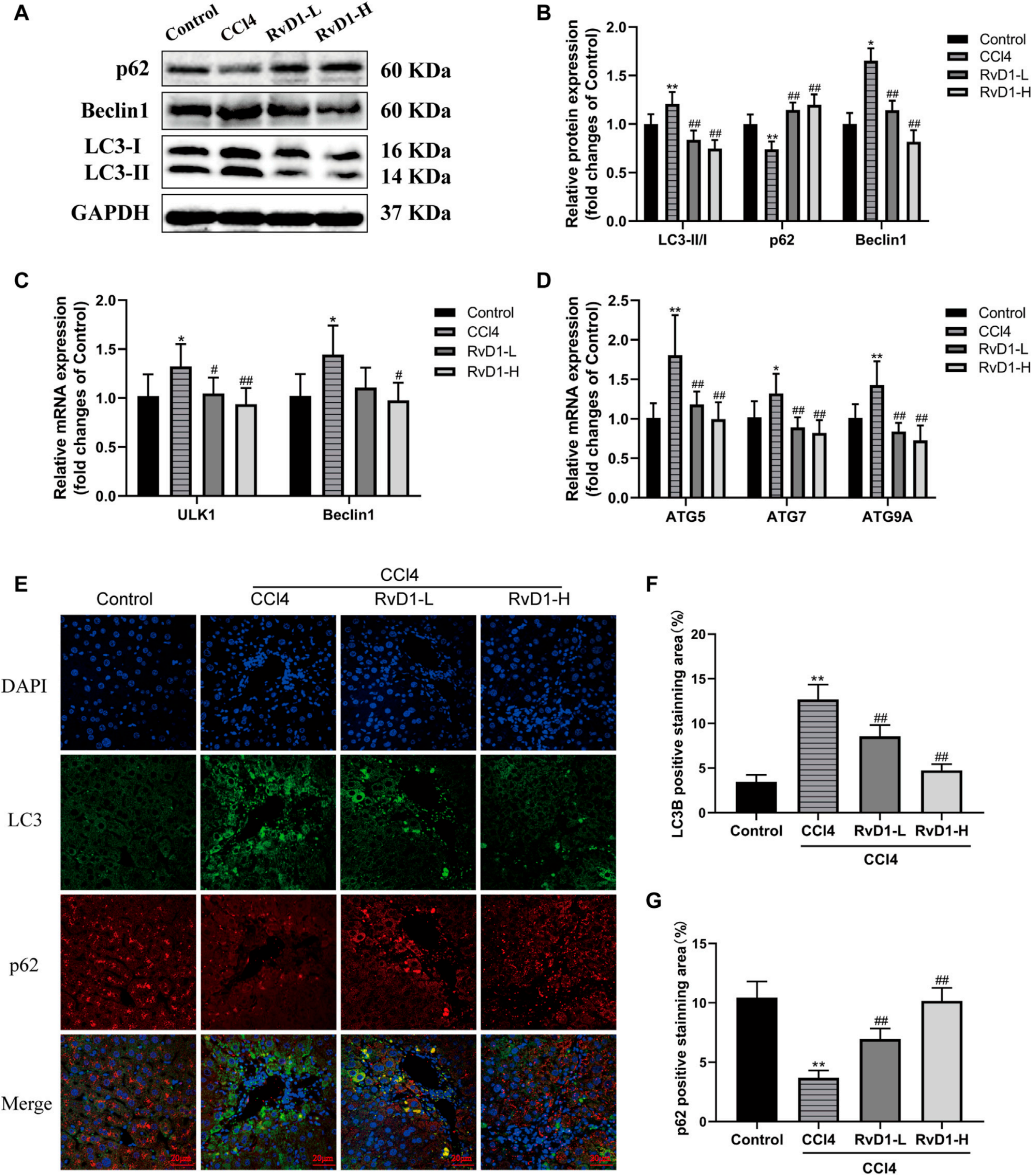
RvD1 alleviated CCl4-induced liver fibrosis in mice by regulating autophagy. **(A and B)** Western blot analysis of total protein levels of LC3, p62, Beclin1 and GAPDH in liver samples from indicated group. Representative bands are shown. **(C and D)** The qRT-PCR analysis of ULK1, ATG5, Beclin1, ATG7, and ATG9A mRNA levels in mouse liver samples. **(E)** Representative images of double immunofluorescent staining against LC3 (red color) and p62 (green color) in liver sections from each group, scale bar = 20 μm. **(F and G)** Semi-quantitative analysis of the number of LC3 and p62 puncta per cell. Data are presented as the mean ± SD, n = 5–6 mice/group. *p < 0.05, **p < 0.01 vs. the Control group; ^#^ *p* < 0.05, ^##^ *p* < 0.01 vs. the CCl4 group.

Studies have shown that autophagy provides energy for HSC activation by consuming intracellular lipids (Hernández-Gea et al., 2012). So, in fibrosis development, autophagic process was significantly induced and the transformation of autophagy marker LC3-I to LC3-II was increased accompanied by the activation of HSC (Tanida et al., 2008). The present results from western blot and immunofluorescence showed that CCl4 intervention resulted in a significant accumulation of LC3-II, but no obvious change was observed in LC3-I, thus the ratio of LC3-II/LC3-I increased, while RvD1 treatment reverse LC3 expression in a dose dependent manner (Figures 5A,B,F). The changes of Beclin1 in groups were consistent with LC3-II (Figures 5A–C). Similarly, the results of qRT-PCR showed that the mRNA levels of these ULK1, ATG5, Beclin1, ATG7, and ATG9A, which are involved in the formation of autophagosomes (Li and Zhang, 2019), were decreased in a dose dependent manner with the intervention of RvD1 (Figures 5C,D). In contrast to the changes in LC3-II, p62, an autophagy specific substrate which is negatively correlated with autophagic flux (Lippai and Lőw, 2014), was decreased under the intervention of CCl4, while the expression of p62 protein was increased following RvD1 treatment (Figures 5A,B,G). These results suggested that RvD1 inhibited autophagic flux in the liver of mice treated with CCl4.

### RvD1 Reduced LX-2 Cell Activation by Suppressing Autophagy

**FIGURE 6:**
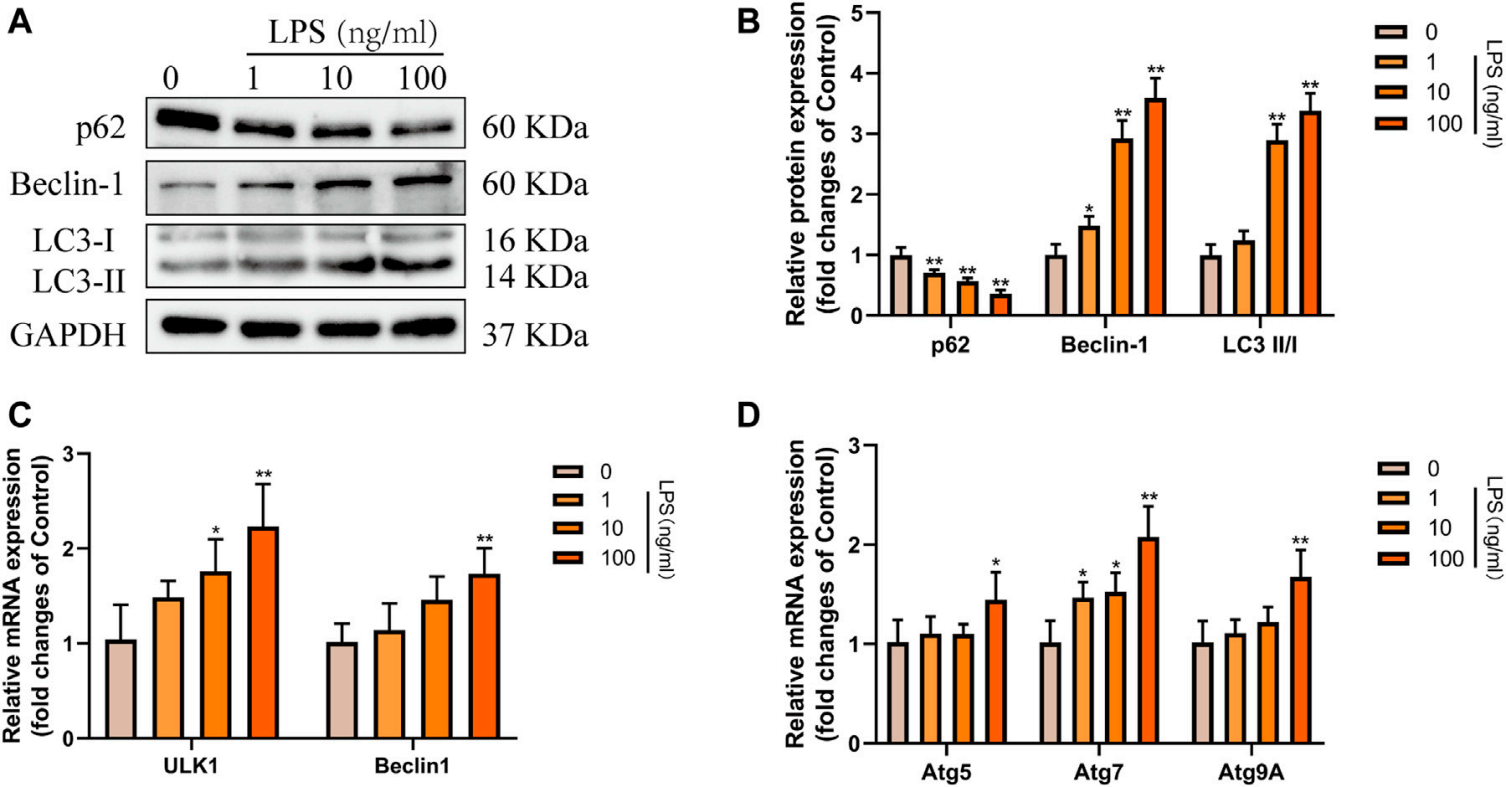
Autophagy was activated in LX-2 cells under LPS stimulation. **(A and B)** Western blot analysis of total protein levels of p62, LC3, Beclin1 and GAPDH in LX-2 cells treated with various concentrations of LPS (1–100 ng/ml) or an equal volume of PBS for 24 h. Representative bands are shown. **(C and D)** The qRT-PCR analysis of ULK1, ATG5, Beclin1, ATG7, and ATG9A mRNA levels in LX-2 cells. At least three independent experiments were carried out. Data are presented as the mean ± SD, n = 4 samples/group. *p < 0.05, **p < 0.01 vs. the Control group.

Subsequently, to determine the effect of RvD1 on autophagy in HSCs *in vitro*. We treated LX-2 cells with different concentrations (0–100 ng/ml) of LPS to induce cell activation. Results of western blot showed that with the increasing concentrations of LPS, the ratio of LC3-II/LC3-I and the expression of Beclin1 were gradually elevated, and the level of autophagy specific substrate p62 protein gradually decreased (Figures 6A,B). In addition, with increasing LPS concentration, the mRNA levels of ULK1, ATG5, Beclin1, ATG7 and ATG9A were also increased to varying degrees (Figures 6C,D). These results indicated that LPS could stimulate the activation of LX-2 cells and induce autophagy. Consistent with the degree of activation of LX-2, the autophagy activation was the most significant when LPS was at 100 ng/ml.

**FIGURE 7:**
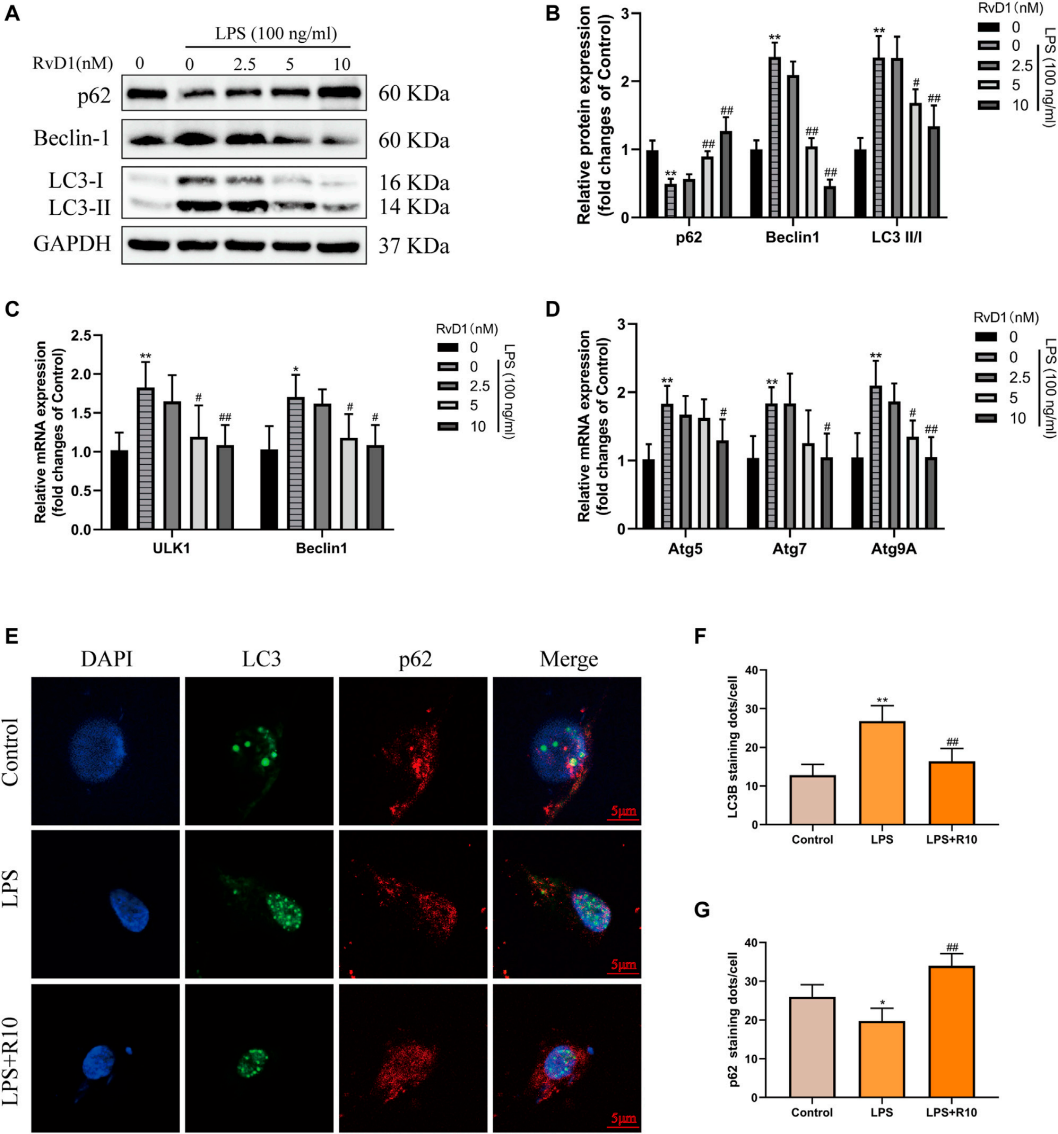
RvD1 reduced LX-2 cells activation by suppressing autophagy. **(A and B)** LX-2 cells were co-treated with the indicated concentrations (2.5–10 nM) of RvD1 and LPS (100 ng/ml) for 24 h. Cells in control group received PBS treatment. Western blot analysis of total protein levels of p62, LC3, Beclin1 and GAPDH in LX-2 cells. **(C and D)** The qRT-PCR analysis of ULK1, ATG5, Beclin1, ATG7, and ATG9A mRNA levels in LX-2 cells. **(E and G)** Representative images of double immunofluorescent staining against LC3 (green color) and p62 (red color) in LX-2 cells, and quantitative analysis of LC3 and p62 fluorescence per cell (8 cells/sample). At least three independent experiments were carried out. Data are presented as the mean ± SD, n = 4 samples/group. *p < 0.05, **p < 0.01 vs. the Control group; ^#^ *p* < 0.05, ^##^ *p* < 0.01 vs. the LPS group.

Then, LX-2 cells were treated with PBS, 100 ng/ml LPS and 100 ng/ml LPS plus different concentrations (2.5, 5, 10 nM) of RvD1, respectively. The expression changes of p62, Beclin1, and LC3 were detected. The results of western blot showed that the expression of p62 inhibited by LPS was elevated with the increasing concentrations of RvD1, while the ratio of LC3-II/I and the expression of Beclin1 promoted by LPS were decreased after RvD1 treatment in a dose-dependent manner (Figures 7A,B). The similar changes of LC3 and p62 were also verified by cell-immunofluorescence staining, which further supported the inhibition of autophagy activation in HSCs by RvD1 (Figures 7E–G). In addition, to detect the dynamic process of autophagy flux, a tandem GFP-mRFP-LC3 plasmid was transfected into LX-2 cells. After 24 h, cells were treated with LPS (100 ng/ml) or/and RvD1 (10 nM) and continued to be cultured for 24 h, the changes of autophagy flux were observed under laser confocal microscopy. The yellow puncta and red puncta indicate autophagosomes and autophagolysosomes, respectively. As shown in Supplementary Figure S2, RvD1 significantly reduced the number of autophagosomes and autophagolysosomes in activated LX-2 cells. Finally, we found that the mRNA levels of multiple ATGs (Beclin1, ULK1, ATG5, ATG7, ATG9A) were decreased in a dose dependent manner upon RvD1 intervention by qRT-PCR (Figures 7C,D). The above results suggested that RvD1 inhibited both autophagosome formation and autophagolysosome degradation in activated LX-2 cells.

### RvD1 inhibited Autophagy in Activated LX-2 Cells by Regulating AKT/mTOR Pathway

**FIGURE 8:**
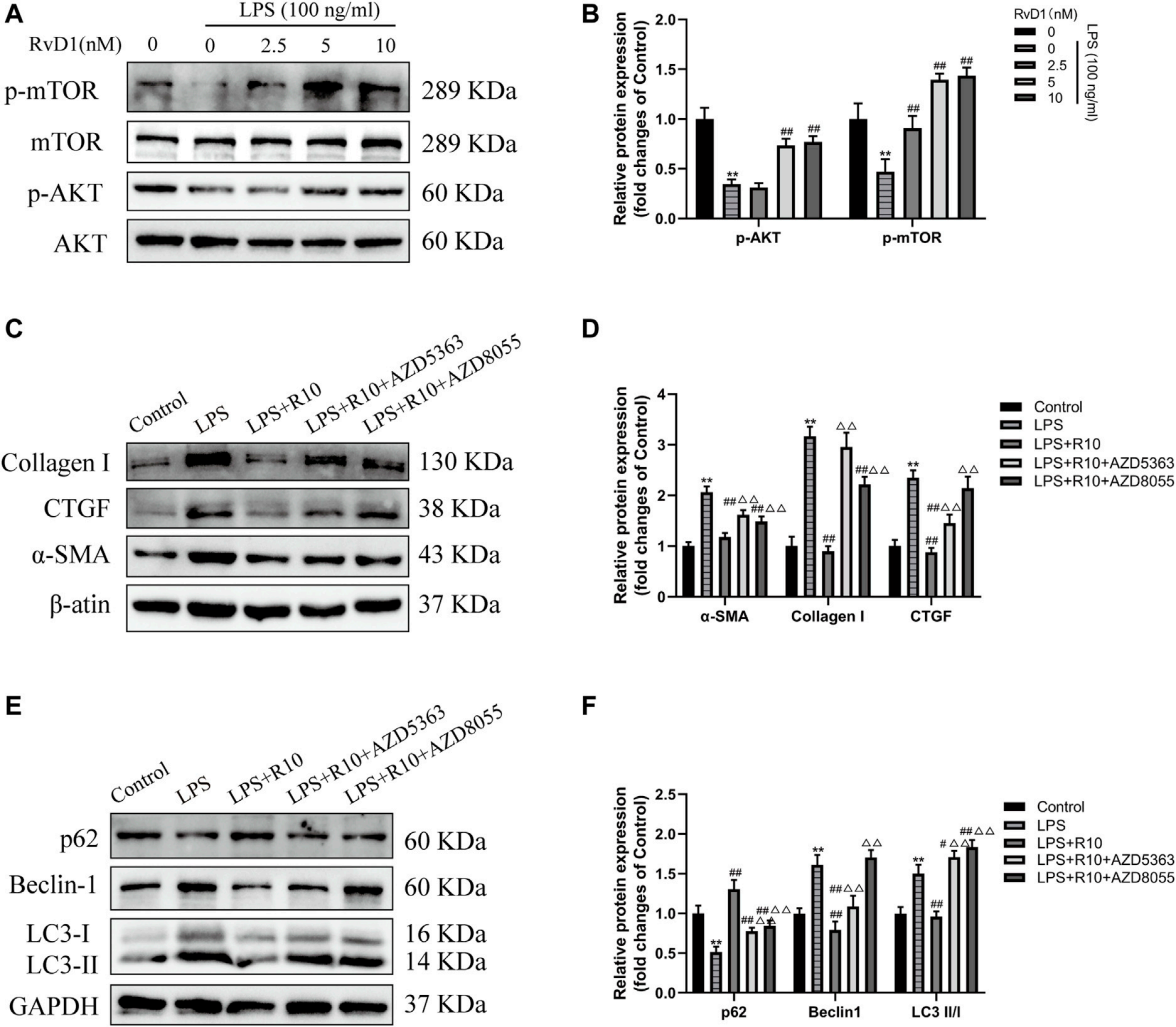
RvD1 inhibited autophagy in activated LX-2 cells by regulating AKT/mTOR pathway. **(A and B)** LX-2 cells were pretreated with AZD5363 (AKT inhibitor, 10 μM) or AZD8055 (mTOR inhibitor, 500 nM), respectively, followed by LPS (100 ng/ml) and RvD1 (10 nM) treatment for 24 h. Western blot analysis of total protein levels of p-AKT, AKT, p-mTOR and mTOR in LX-2 cells. **(C and D)** Western blot analysis of total protein levels of α-SMA, Collagen I and CTGF in LX-2 cells. Cells in LPS + R10 group received 10 nM RvD1 and 100 ng/ml LPS treatments for 24 h **(E and F)** Western blot analysis of LC3, p62 and Beclin1 protein levels in LX-2 cells. Cells in LPS + R10 group received 10 nM RvD1 and 100 ng/ml LPS treatments for 24 h. At least three independent experiments were carried out. Data are presented as the mean ± SD, n = 4 samples/group. *p < 0.05, **p < 0.01 vs. the Control group; ^#^ *p* < 0.05, ^##^ *p* < 0.01 vs. the LPS group; ^△^ *p* < 0.05, ^△△^ *p* < 0.01 vs. the LPS + R10 group.

AKT/mTOR is a major signaling pathway regulating autophagy (Noguchi et al., 2014; Wang and Zhang, 2019). To clarify whether RvD1 inhibits autophagy in LX-2 cells through AKT/mTOR pathway. The activated LX-2 cells were treated with different concentrations (2.5, 5, 10 nM) of RvD1 for 24 h, and then the protein levels of related indicators were detected by western blot. The results showed that RvD1 significantly elevated the expression of phosphorylated proteins of AKT and m-TOR in a dose dependent manner, namely, both the ratio of p-AKT/AKT and p-mTOR/mTOR increased (Figures 8A,B).

To further validate the effect of RvD1 on AKT/mTOR signaling pathway, LX-2 cells were pretreated with AZD5363 (AKT inhibitor, 10 μM) or AZD8055 (mTOR inhibitor, 500 nM), respectively, followed by LPS (100 ng/ml) and RvD1 (10 nM) treatment. The results showed that the protein levels of α-SMA, Collagen I and CTGF were significantly increased after AZD5363 or AZD8055 intervention, compared with the LPS + R10 group by western blot (Figures 8C,D). Next, we continued to evaluate changes in autophagy by detecting the protein expression of LC3, p62 and Beclin1, and found that AZD5363 and AZD8055 intervention could promote the elevation of p62 protein, but reduce the protein expression of LC3 and Beclin1 (Figures 8E,F). The above results reversely verified that AZD5363 and AZD8055 antagonized the inhibition effect of RvD1 on autophagy in activated HSCs.

## Discussion

Due to lacking effective anti-fibrotic therapies, liver fibrosis and cirrhosis has been a severe global health burden and accounts for up to 2% of all deaths worldwide (Mokdad et al., 2014). It is urgent to seek appropriate drugs to tackle this public health dilemma. In this study, for the first time, we demonstrate that RvD1 is a potential pharmacotherapy candidate for relieving liver injury and fibrosis. Our study also shows that the anti-fibrotic effect of RvD1 is partly mediated by inhibiting autophagy in activated HSCs through AKT/mTOR pathway both in CCl4-treated mice and in LPS-induced LX-2 cells.

As is known to all, liver fibrosis generates from chronic liver injury induced by various factors, including excessive fat deposition, drug and toxicant damage, viral infection, biliary tract diseases, autoimmune disorder, and congenital or genetic anomalies (Roehlen et al., 2020). The corresponding animal models are usually used to study liver fibrosis of different etiologies. CCl4, a known hepatotoxin, has been demonstrated to generate amounts of free radical species under the catalysis of cytochrome P450 enzymes, causing massive hepatocyte necrosis (Dai et al., 2018). In addition, under the stimulation of CCl4 and its metabolites, the injured hepatocytes and activated macrophages secrete massive inflammatory factors and pro-fibrogenic factors to activate HSCs and aggravate liver fibrosis (Liu et al., 2019). Long-term practice has shown that CCl4 intervention has satisfactory repeatability and is suitable for mimicking human liver diseases (Fujii et al., 2010; Dai et al., 2018). CCl4 intoxication inflicts liver damage and fibrosis in a dose-and time-dependent manner. Four-week repeated injection of CCl4 could induce early hepatic fibrosis in mice, and 6–8 weeks of CCl4 intervention may cause more typical pathological changes in mice liver. Moreover, the course of disease beyond 12 weeks led to the development of cirrhosis in mice with high mortality. Based on previous literatures (Shrestha et al., 2016; Liu et al., 2019; Wu et al., 2020), we established a 6 week-CCl4 mice model to determine the effect of RvD1 in liver fibrosis. Histological evaluation and serum transaminase results showed that RvD1 exerted positive effect in alleviating liver injury and fibrosis.

HSCs activation is a crucial step in liver fibrogenesis. LPS was widely used to induce the activation of LX-2 cells to establish *in vitro* model of hepatic fibrosis. As a inflammatory substance, LPS is mostly produced in the cell wall of gram-negative bacteria and is often used to induce various inflammatory damage models (Barabutis et al., 2019; Hui et al., 2020). Previous studies have shown that in the development process of liver fibrosis, LPS can indirectly or directly stimulate the activation of HSCs and induce the expressions of ECM components, which may involve TLR4/NF-κB, autophagy and other signaling pathways (Soares et al., 2010; Chen et al., 2017). LX-2 is a class of immortalized HSCs derived from normal human liver tissue, which is characterized by the expression of α-SMA and the production of cytokines and collagen after being activated (Xu et al., 2005). As demonstrated by CCK-8 and western blot, our results showed that 100 ng/ml LPS significantly promoted LX-2 activation and autophagy in cells, which is consistent with previous study.

Autophagy is an evolutionarily conserved cellular process that occurs to maintain cellular homeostasis by degrading damaged organelles, abnormal protein aggregates and damaged DNA in lysosomes (Yu et al., 2018). In liver fibrosis, autophagy has been reported to play bidirectional regulatory role in the occurrence and development of hepatic fibrosis. Moderate autophagy promotes liver fibrosis. Augmenting bodies of evidences have revealed that autophagy is abnormally active in both patients and rodents with chronic fibrotic liver disease (Hernández-Gea et al., 2012; Li et al., 2018). It is more interesting to note that autophagy is increased with concomitant decreased intracellular lipid contents during the process of HSCs activation, which means autophagy induced in HSCs may serve as a vital energy source to fuel the activation and proliferation of itself by breaking down intracellular lipids (Hernández-Gea et al., 2012). However, excessive autophagy may be beneficial for alleviating liver fibrosis. Chen et al. (2018) proved that HSCs were damaged by excessive autophagy, leading to cell senescence, decreased cell viability and reduced ECM secretion. LC3 and p62 are currently known to be crucial genes regulating autophagy and considered as biomarkers of autophagy flux (Lippai and Lőw, 2014; Runwal et al., 2019). After autophagy is initiated, LC3-I (a cytosolic form of LC3) is transformed into LC3-II (LC3-phosphatidylethanolamine conjugate) and then recruited to autophagosomal membranes, which is essential in the formation of autophagosome (Tanida et al., 2008). As a substrate for LC3-II, the multifunctional ubiquitin-adaptor p62 is concomitantly degraded along with cell contents (Lippai and Lőw, 2014). In this experiment, with the activation of LX-2 cells and the aggravation of liver fibrosis in mice, the expression of LC3-II gradually increased, while the expression of p62 gradually decreased, indicating that the level of autophagy activation was consistent with HSC activation. Therefore, these results of our article supports the theory that autophagy induces HSCs activation and promotes liver fibrosis.

As demonstrated by cerulein-induced acute pancreatitis model, RvD1 could regulate autophagy (Wang et al., 2018), but whether RvD1 affects HSCs activation through mediating autophagic process remains unknown. After RvD1 intervention, expression of p62 both *in vivo* and *in vitro* were increased, and the turnover of LC3-I to LC3-II were inhibited, suggesting that the protective effect of RvD1 against Liver fibrosis was ascribed to the inhibition of autophagy. It is worth mentioning that the increased expression of LC3-II may be owing to the autophagosome accumulation at the earliest phage, or the impaired clearance of the autophagolysosome in the late phase (Tanida et al., 2008). Autophagolysosome generates from the autophagosome and lysosome fusion. Using differential performance of GFP (green fluorescent protein) and mRFP (red fluorescent protein) under the acidic conditions of lysosomal formation, GFP-mRFP-LC3 double plasmid was widely used to trace the morphological alterations of autophagic flux. We observed that the formation of both autophagosome and autophagolysosome in LX-2 cells was significantly reduced in the RvD1 intervention group, indicating that RvD1 blocked the initiation stage of autophagy and impaired autophagic flux in HSCs.

It is demonstrated that AKT/mTOR pathway negatively regulates autophagy and the inhibition of AKT/mTOR thus activates autophagy (Wang and Zhang, 2019). AKT, as a phosphorylate key molecules, is involved in the inhibition of autophagosome formation and lysosomal accumulation by binding to Phafin2, which is a core lysosomal protein to locate lysosomes (Noguchi et al., 2014). As downstream transduction of the AKT signaling pathway, mTOR (target of rapamycin) plays a master role in modulating autophagy, lysosomal biogenesis, and cell growth and metabolism (Ersahin et al., 2015; Wang and Zhang, 2019). In our study, RvD1 dose-dependently promoted the expression of p-Akt and p-mTOR in activated LX-2 cells, which was consistent with the changes of autophagy markers. While after the administration of AKT inhibitor (AZD5363) and mTOR inhibitor (AZD8055), the expression of p62 was decreased, LC3-II/I ratio was increased, indicating that RvD1 can inhibit autophagy of HSC by activating AKT/mTOR signaling pathway.

Despite many meaningful findings of this study, limitations still exist. In the present work, we identify that RvD1 exerts anti-fibrosis effects by suppressing autophagy activity in HSCs. However, a 6-week of CCl4 intraperitoneal injection is not enough to induce advanced liver cirrhosis. Further work may be needed to fully elucidate the effects of RvD1 on liver fibrosis at advanced stage. Secondly, it is remain to be verified whether RvD1 shows a similar effect on liver fibrosis/cirrhosis caused by other etiologies, such as alcohol, viral infection and so on. Finally, since various cells are involved in the development of liver fibrosis, the anti-fibrotic effect of RvD1 may not only act on HSCs, but also affect other cells. Since the activation of HSCs is the core mechanism of hepatic fibrosis, our *in vitro* study mainly focuses on HSCs. The effect of RvD1 on other cells and its relationship with HSCs also may be worth further investigation.

In conclusion, our data prove that RvD1 inhibits HSCs autophagy by regulating AKT/mTOR signaling pathway, which reduces HSCs activation and blocks the occurrence and development of liver fibrosis. RvD1 treatment is expected to become a novel therapeutic strategy against liver fibrosis and other chronic fibrotic disease.

## Data Availability

The raw data supporting the conclusions of this article will be made available by the authors, without undue reservation.
