# Supplementary material for: Resolvin D1 attenuates CCl4 Induced Liver Fibrosis by Inhibiting Autophagy-Mediated HSC activation via AKT/mTOR Pathway
Source: Front Pharmacol. 2021 Dec 20;12:792414. doi: 10.3389/fphar.2021.792414 (PMC8721195; doi:10.3389/fphar.2021.792414)
Supplement: Supplementary file 1 [file DataSheet1.docx]

**Table S1**. Specific primary antibodies used in Western blotting assay.

| Primary antibody | Brand and Catalog number | Dilution |
| --- | --- | --- |
| GAPDH | ABclonal (AC002) | 1:10000 |
| β-actin | Proteintech (20536-1-AP) | 1:8000 |
| CTGF | ABclonal (A11067) | 1:1000 |
| α-SMA | ABclonal (A2319) | 1:4000 |
| Collagen I | Proteintech (14695-1-AP) | 1:2000 |
| Beclin1 | ABclonal (A7353) | 1:1000 |
| LC3B | Sigma (L7543) | 1:2000 |
| p62 | Proteintech (55274-1-AP) | 1:4000 |
| mTOR | CST (2972) | 1:1000 |
| p-mTOR | CST (2971) | 1:1000 |
| p-AKT | CST (4060) | 1:1000 |
| AKT | CST (4691) | 1:1000 |

**Table S2.** Primer sequences used for qRT-PCR.

| Gene | Species | Sequence (5'->3') |
| --- | --- | --- |
| GAPDH | Mus musculus | Forward AGGTCGGTGTGAACGGATTTG |
|  |  | Reverse TGTAGACCATGTAGTTGAGGTCA |
| α-SMA | Mus musculus | Forward GTCCCAGACATCAGGGAGTAA |
|  |  | Reverse TCGGATACTTCAGCGTCAGGA |
| CTGF | Mus musculus | Forward GGGCCTCTTCTGCGATTTC |
|  |  | Forward ATCCAGGCAAGTGCATTGGTA |
| Collagen I | Mus musculus | Forward GCTCCTCTTAGGGGCCACT |
|  |  | Reverse CCACGTCTCACCATTGGGG |
| TIMP-1 | Mus musculus | Forward CGAGACCACCTTATACCAGCG |
|  |  | Reverse ATGACTGGGGTGTAGGCGTA |
| Vimentin | Mus musculus | Forward TCCACACGCACCTACAGTCT |
|  |  | Reverse CCGAGGACCGGGTCACATA |
| Beclin1 | Mus musculus | Forward ATGGAGGGGTCTAAGGCGTC |
|  |  | Reverse TGGGCTGTGGTAAGTAATGGA |
| ATG9A | Mus musculus | Forward CAGTTTGACACTGAATACCAGCG |
|  |  | Reverse AATGTGGTGCCAAGGTGATTT |
| ATG5 | Mus musculus | Forward TGTGCTTCGAGATGTGTGGTT |
|  |  | Reverse GTCAAATAGCTGACTCTTGGCAA |
| ATG7 | Mus musculus | Forward GTTCGCCCCCTTTAATAGTGC |
|  |  | Reverse TGAACTCCAACGTCAAGCGG |
| ULK1 | Mus musculus | Forward TGGAGGTGGCCGTCAAATG |
|  |  | Reverse CGCATAGTGTGCAGGTAGTC |
| GAPDH | Homo sapiens | Forward CTGGGCTACACTGAGCACC |
|  |  | Reverse AAGTGGTCGTTGAGGGCAATG |
| α-SMA | Homo sapiens | Forward CTATGAGGGCTATGCCTTGCC |
|  |  | Reverse GCTCAGCAGTAGTAACGAAGGA |
| CTGF | Homo sapiens | Forward ACCGACTGGAAGACACGTTTG |
|  |  | Reverse CCAGGTCAGCTTCGCAAGG |
| Collagen I | Homo sapiens | Forward GAGGGCCAAGACGAAGACATC |
|  |  | Reverse CAGATCACGTCATCGCACAAC |
| TIMP-1 | Homo sapiens | Forward ACCACCTTATACCAGCGTTATGA |
|  |  | Reverse GGTGTAGACGAACCGGATGTC |
| Vimentin | Homo sapiens | Forward GACGCCATCAACACCGAGTT |
|  |  | Reverse CTTTGTCGTTGGTTAGCTGGT |
| Beclin1 | Homo sapiens | Forward GGTGTCTCTCGCAGATTCATC |
|  |  | Reverse TCAGTCTTCGGCTGAGGTTCT |
| ATG9A | Homo sapiens | Forward TGTTTCTCAATGAATGGAGCCTC |
|  |  | Reverse AAGTTAGCGATGCCAATCCAC |
| ATG5 | Homo sapiens | Forward AAAGATGTGCTTCGAGATGTGT |
|  |  | Reverse CACTTTGTCAGTTACCAACGTCA |
| ATG7 | Homo sapiens | Forward CAGTTTGCCCCTTTTAGTAGTGC |
|  |  | Reverse CCAGCCGATACTCGTTCAGC |
| ULK1 | Homo sapiens | Forward GGCAAGTTCGAGTTCTCCCG |
|  |  | Reverse CGACCTCCAAATCGTGCTTCT |


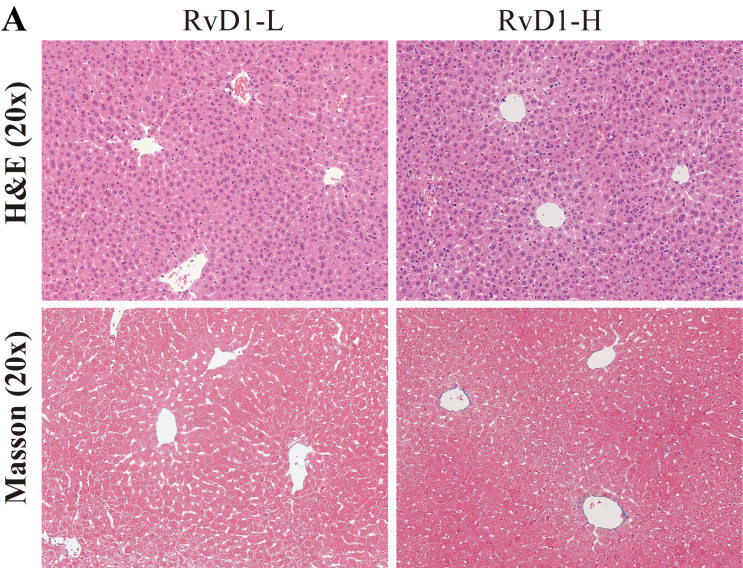


**FIGURE S1**┃ RvD1 treatment alone did not change normal pathological morphology of liver. (A) Representative images of H&E and Masson staining in liver samples from RvD1-l and RvD1-H groups (magnification: 20×, scale bar: 200 μm, n = 3 mice/group).


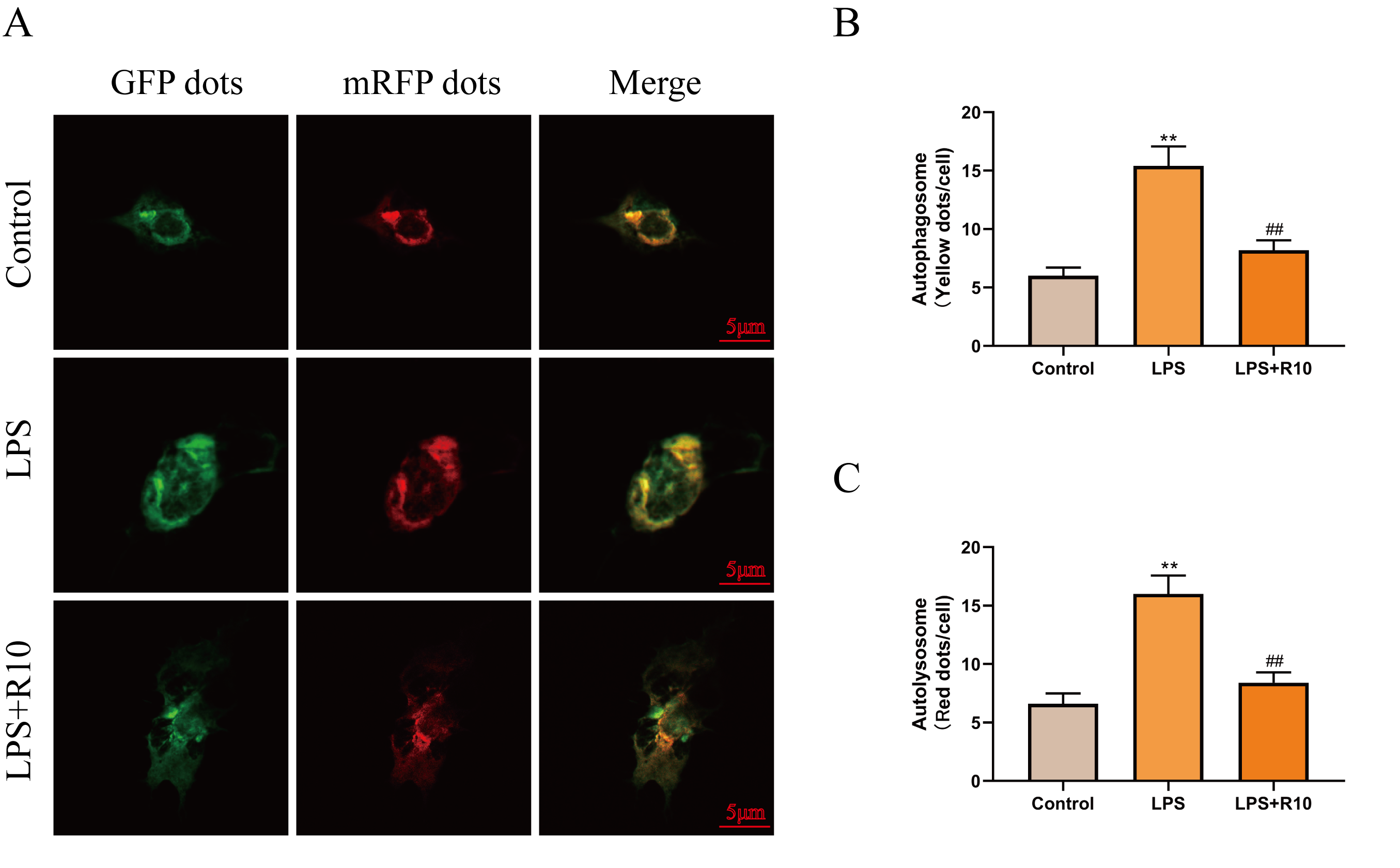


**FIGURE S2**┃ Using GFP-mRFP-LC3 double plasmid to monitor the dynamic changes of autophagy. (A) LX-2 cells were transfected with the GFP-mRFP LC3 plasmid for 24 h, followed by the co-treatment of LPS and RvD1. Representative images of fluorescent GFP-LC3 puncta, mRFP-LC3 puncta, and merged images are shown. Scale bar=5 μm. (B-C) Quantification of autolysosomes (red puncta) and autophagosomes (yellow puncta) in merged images per cell (8 cells/independent experiment). Cells in LPS+R10 group received 10 nM RvD1 and 100 ng/ml LPS treatments for 24 h. At least three independent experiments were carried out. Data are presented as the mean ± SD, n = 4 samples/group. *P < 0.05, **P < 0.01 versus the Control group; ^#^P < 0.05, ^##^P < 0.01 versus the LPS group.
